# The *MMP-8* rs11225395 Promoter Polymorphism Increases Cancer Risk of Non-Asian Populations: Evidence from a Meta-Analysis

**DOI:** 10.3390/biom9100570

**Published:** 2019-10-04

**Authors:** Jiarong Feng, Yudi Chen, Wenxi Hua, Xiaohan Sun, Yanjie Chen, Yu Liu, Jiaying Fan, Yuening Zhao, Lixiang Zhao, Xiaojing Xu, Xiaoqin Yang

**Affiliations:** 1 Department of Bioinformatics, School of Biology and Basic Medical Sciences, Soochow University, 199 Ren’ai Road, Suzhou 215123, Chinachenyudicamile@hotmail.com (Y.C.); sxh15370341176@hotmail.com (X.S.); chenyanjie6593@163.com (Y.C.);; 2 Medical College, Soochow University, 199 Ren’ai Road, Suzhou 215123, Chinazhaoyuening1629@outlook.com (Y.Z.); 3 Department of Biotechnology, School of Biology and Basic Medical Sciences, Soochow University, 199 Ren’ai Road, Suzhou 215123, China; kaiifan1110@outlook.com; 4 Department of Immunology, School of Biology and Basic Medical Sciences, Soochow University, 199 Ren’ai Road, Suzhou 215123, China; 5 Department of Cell Biology, School of Biology and Basic Medical Sciences, Soochow University, 199 Ren’ai Road, Suzhou 215123, China

**Keywords:** MMP-8, single-nucleotide polymorphism, cancer, meta-analysis

## Abstract

This meta-analysis aimed to systematically review the evidence on cancer risk of the *MMP-8* rs11225395 promoter polymorphism. Relevant studies published by 12 June 2019 were identified by systematically searching PubMed, Web of Science, Cochrane Library, CNKI and Wanfang databases. R programs and STATA software were used to calculate odds ratio (OR) and 95% confidence interval (CI). In total, 7375 cancer samples and 8117 controls were included by integrating 15 case-control data sets. Pooled estimates from the statistical analysis revealed no statistical significance for the association between this polymorphism and cancer risk. All pooled estimates resulting from subgroup analyses by cancer type and sample size were not materially altered and did not draw significantly different conclusions. The stratified analyses according to geographic region showed the statistical significance for increased cancer risk of the *MMP-8* rs11225395 polymorphism in non-Asian populations under the allele model (OR = 1.11, 95% CI: 1.04–1.19), homozygote model (OR = 1.22, 95% CI: 1.05–1.41), heterozygote model (OR = 1.21, 95% CI: 1.07–1.36), and dominant model (OR = 1.21, 95% CI: 1.08–1.35). However, no statistical significance was detected in Asian populations. In conclusion, these findings suggested that the *MMP-8* rs11225395 polymorphism is associated with elevated susceptibility to cancer in non-Asian populations.

## 1. Introduction

Cancer is still one of the most devastating diseases, leading to millions of deaths worldwide each year. As a multifactorial disease, this life-threatening malignancy could result from lifestyle factors, dietary habits, environmental impact, and genetic predispositions. Its relevance to genome variations, including single-nucleotide polymorphism (SNP), has been backed up with more and more scientific evidence [1,2,3,4].

Matrix Metallopeptidase 8 (MMP-8) gene, located on chromosome 11q22.2, encodes a collagenase participating in the process of extracellular matrix degradation and remodeling, whose deregulation could promote tumorigenesis and progression. Although several previous studies have reported the inhibitory effect of the MMP-8 gene on carcinogenesis and metastasis, conflicting evidence for its function as an oncogene also exists. In oral tongue squamous cell carcinoma, high MMP-8 expression could reduce tumor invasion and migration [5] and lead to improved survival [6]. In breast cancer, MMP-8 may affect the metastatic potential through inhibition against lymph node metastasis [7,8]. However, in ovarian cancer, the overexpression of MMP-8 could promote the invasive potential of cancer [9], and in both hepatocellular carcinoma [10] and colorectal cancer [11], higher MMP-8 serum levels have been reported to be associated with significantly worse survival.

The *MMP-8* rs11225395 (C-799T) polymorphism could lead to a C to T single-nucleotide variation in the promoter region. Scattered evidence illustrated that the T allele of this polymorphism could lead to significantly higher promoter activity and protein expression than its C allele [12,13,14]. Prognostic analyses suggested that the T allele predicts better overall survival among patients with early-stage breast cancer [12,15], but the opposite report for ovarian cancer has also been presented [16]. Based on the crucial roles of the MMP-8 gene and the prognostic impact of this polymorphism, it is imperative to determine the association between the rs11225395 polymorphism and cancer risk.

Discrepant results for the association between the *MMP-8* rs11225395 polymorphism and cancer risk have been reported. The limited sample size of all these studies may also markedly reduce the statistical power of their conclusions. To better elucidate the genetic impact of the *MMP-8* rs11225395 polymorphism, these scattered case-control studies should be pooled into a meta-analysis.

## 2. Materials and Methods

### 2.1. Data Sources and Identification

Potentially eligible studies were queried in five literature databases: PubMed (https://www.ncbi. nlm.nih.gov/pubmed), Clarivate Web of Science (https://www.webofknowledge.com/), Cochrane Library (https://www.cochranelibrary.com/advanced-search), CNKI (http://www.cnki.net/), and Wanfang Data (http://www.wanfangdata.com.cn/). The retrieval type consists keywords for polymorphism ID, gene name and symbol (“MMP8”, “MMP-8”, “matrix metalloproteinase-8”, and “rs11225395”), aliases for cancer (“neuroblastoma”, “melanoma”, “lymphoma”, “osteosarcoma”, “leukemia”, “tumor”, “cancer”, “carcinoma” and “adenocarcinoma”), and terms representing single-nucleotide polymorphism (“polymorphism”, “SNP”, and “variant”). Articles citing the studies involved in this meta-analysis were also retrieved by Google Scholar (https://scholar. google.com/). The references of the eligible articles and reviews were manually examined to identify additional relevant studies. Results retrieved from the inception of these databases up to 12 June 2019 were considered for this meta-analysis.

### 2.2. Study Eligibility Evaluation and Data Extraction

To evaluate the eligibility of each study, four authors independently screened the list of identified literature by sticking to the same inclusion and exclusion criteria. For a study to be excluded from further analysis, the full-text context should be accessible, and the study had to meet at least one of the following prespecified exclusion criteria: (a) not for the *MMP-8* rs11225395 promoter polymorphism; (b) not for cancer risk; (c) no genotype data for cancer and control samples. Inclusion criteria were as follows: (a) enough data for pooled estimates in at least one genetic model; (b) no benign tumor samples were included in the case group; (c) in Chinese or English.

Four authors independently performed the data extraction. The following information regarding study design features and patient characteristics were recorded: (a) name of the first author, (b) year of publication, (c) cumulated genotype number in cancer and control groups, (d) geographic region of involved samples, (e) sample size, (f) genotyping method, (g) cancer type. Additionally, appropriateness of the Hardy–Weinberg equilibrium (HWE) in the control group was statistically measured (*p* < 0.05 indicates statistical significance) and then qualitatively labeled as a study characteristic. The Newcastle–Ottawa Scale (NOS) system was applied to evaluate the quality of the studies involved (http://www.ohri.ca/programs/clinical_epidemiology/nosgen.pdf). A study scoring greater than or equal to seven out of nine will be labeled with ‘high quality’, four to six as ‘medium quality’ and less than four as ‘poor quality’.

An in-house discussion was held to resolve all disagreement about the eligibility of a single study or difference among the sets of information extracted from involved studies and reach an entire consensus.

### 2.3. Statistics Analysis

Two R (version: 3.5.1, http://cran.r-project.org/) programmers were delegated to develop statistical analysis scripts for statistics analysis. Moreover, two STATA software (version 14.2, STATA Corporation, College Station, TX, USA) operators were also appointed to validate the results. Pooled odds ratio (OR) and 95% confidence interval (95% CI) were calculated to quantitatively assess the cancer risk in the allele model (T vs. C), homozygote model (TT vs. CC), heterozygote model (CT vs. CC), dominant model (CT+TT vs. CC), and recessive model (TT vs. CC+CT). A chi-squared Q-test was performed to detect the heterogeneity among studies (*p* < 0.10 was considered representative of statistically significant heterogeneity). If significant between-study heterogeneity was identified, data were pooled using the DerSimonian–Laird algorithm (random effects model) [17]. Otherwise, the Mantel–Haenszel algorithm (fixed effect model) [18] was applied. The overall population was stratified according to sample size (greater than 500 or not), region (Asia or others), and tumor site (bladder cancer, breast cancer, digestive system cancer, or others). Galbraith plot analysis was used to identify the source of between-study heterogeneity [19]. Publication bias in this meta-analysis was assessed according to the asymmetry of the funnel plot. Egger’s regression asymmetry test [20] and Begg’s adjusted rank correlation test [21] were used to statistically evaluate the degree of asymmetry (if *p* was less than 0.05, publication bias was considered to be of statistical significance). A leave-one-out sensitivity analysis was conducted to evaluate the statistical stableness.

All processes in this meta-analysis strictly adhered to the Preferred Reporting Items for Systematic Reviews and Meta-Analyses (PRISMA) guidelines [22].

## 3. Results

### 3.1. Characteristics of Included Studies

**Figure 1:**
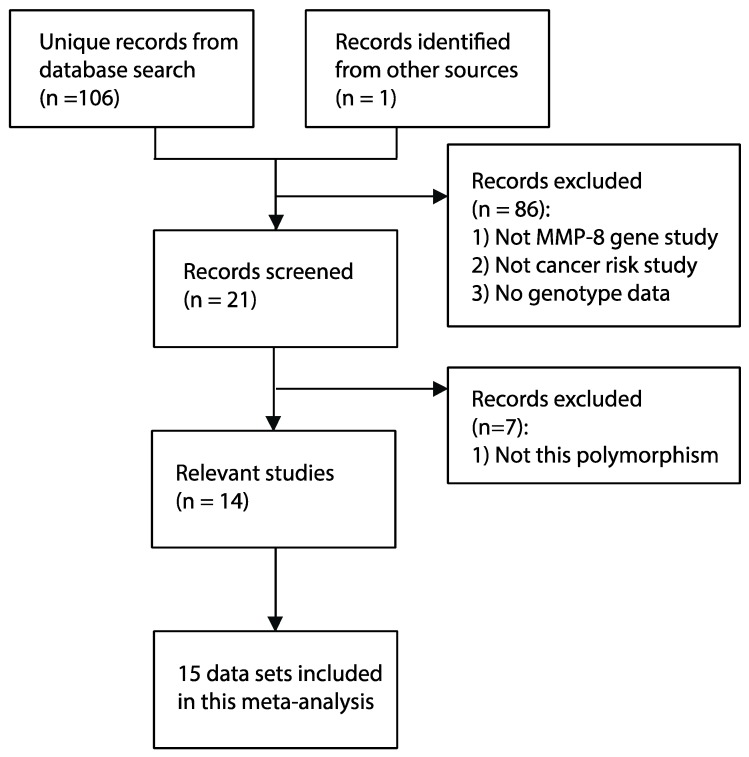
Systematic review flowchart of this meta-analysis.

The workflow for the literature screen and data selection fully revealed our rigid adherence to the PRISMA guidelines (Figure 1). A systematic literature search in five databases found 106 unique manuscripts. Additionally, one breast cancer study [23] citing the article for melanoma risk [13] was identified in citation analysis by Google Scholar. This study for breast cancer risk contained two different cohorts from Poland and the United Kingdom [23]. By implementing the predefined exclusion and inclusion criteria, 15 independent data sets were identified [13,16,23,24,25,26,27,28,29,30,31,32,33,34]. In total, 7375 cancer samples and 8117 controls were included. The characteristics of 15 data sets involved in our meta-analysis are summarized in Table 1. Specifically, the evaluation for methodological quality revealed that 14 of the data sets included are of moderate or high quality. Summarized genotype data was shown in Table 2. The T allele frequencies in the control group of the Asian and non-Asian subdivisions were generally consistent with those reported in several previous genomic studies with large populations (https://www.ncbi.nlm.nih.gov/snp/rs11225395#frequency_tab), such as the Population Architecture using Genomics and Epidemiology study (PAGE, http://www.pagestudy.org/), the 1000 genome project (1000 G) [35], and the genome Aggregation Database (gnomAD, https://gnomad.broadinstitute.org/).

### 3.2. Main Analysis Results

A random effects model was applied in the allele model, homozygote model, dominant model, and recessive model because significant between-study heterogeneity was identified. The observed overlaps between the vertical line showing the null hypothesis (OR = 1) and the 95% CIs for pooled estimates indicated no statistical significance for altered cancer risk (Table 3, allele model: OR = 1.03, 95% CI: 0.95–1.11; homozygote model: OR = 1.01, 95% CI: 0.85–1.20; heterozygote model: OR = 1.06, 95% CI: 0.98–1.14; dominant model: OR = 1.04, 95% CI: 0.93–1.16; recessive model: OR = 0.97, 95% CI: 0.83–1.13).

**Figure 2:**
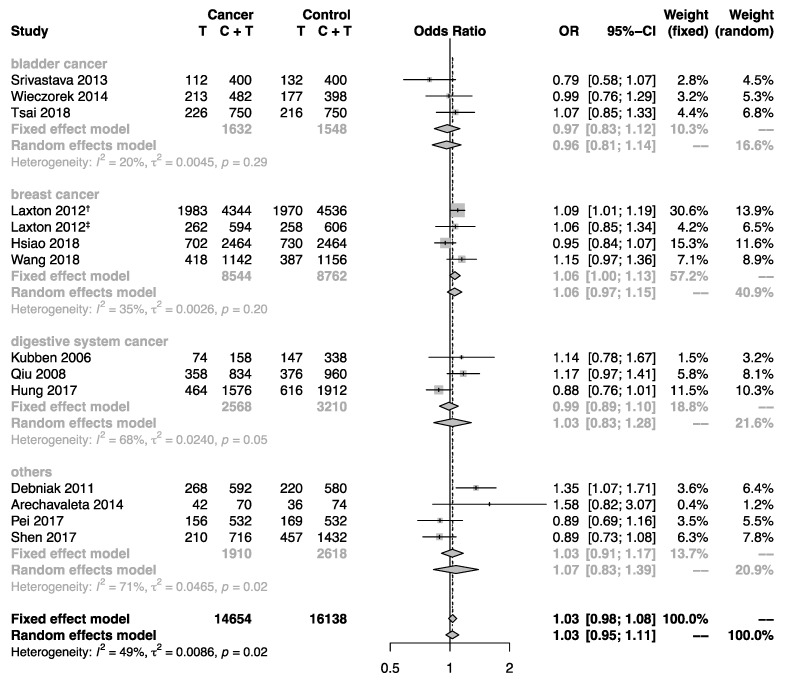
Forest plot showing the association between the *MMP-8* rs11225395 polymorphism and cancer risk in subgroup analysis by cancer type. The pooled estimates were calculated under the allele model. The size of each gray square is proportional to the weight calculated under the fixed effect model, a black dot in a box indicates the odds ratio (OR), and black lines on either end mark the corresponding 95% confidence interval (CI). The gray diamonds represent the overall summary estimate, with 95% CI represented by their widths. The dotted vertical line and the dashed one indicate the pooled ORs calculated by the random effects model and fixed effect model, respectively. The black unbroken vertical line marks the null hypothesis (OR = 1). † marks the population of the United Kingdom in Laxton’s study (2012), and ‡ marks the population of Poland in Laxton’s study (2012).

**Figure 3:**
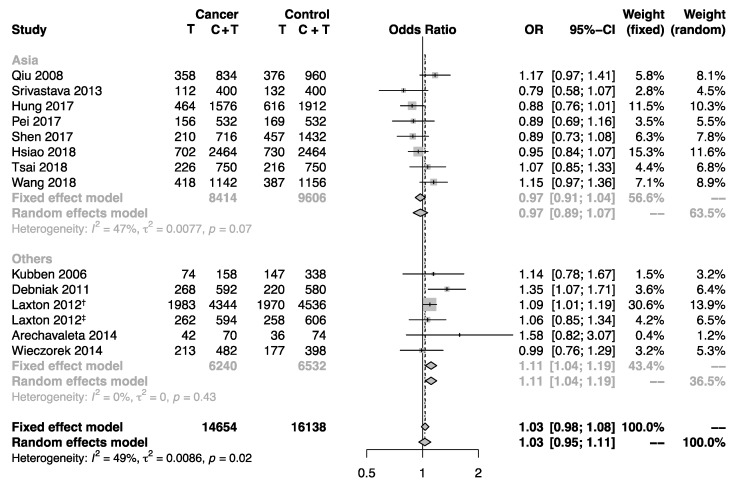
Forest plot showing the association between the *MMP-8* rs11225395 polymorphism and cancer risk in subgroup analysis by region. The pooled estimates were calculated under the allele model. The size of each gray square is proportional to the weight calculated under the fixed effect model, a black dot in a box indicates the odds ratio (OR), and black lines on either end mark the corresponding 95% confidence interval (CI). The gray diamonds represent the overall summary estimate, with 95% CI represented by their widths. The dotted vertical line and the dashed one indicate the pooled ORs calculated by the random effects model and fixed effect model, respectively. The black unbroken vertical line marks the null hypothesis (OR = 1). † marks the population of the United Kingdom in Laxton’s study (2012), and ‡ marks the population of Poland in Laxton’s study (2012).

Subgroup analyses by sample size, cancer type, and region were performed to explore the impact of these factors that may influence the interpretation of the pooled estimates. Subgroups for case-control studies with large and small sample size indicated no substantial difference. Furthermore, the overall population was stratified by cancer type. However, no statistical significance for the association between this polymorphism and cancer risk was detected (Figure 2). Interestingly, the stratifying analysis according to region revealed significantly increased cancer risk for non-Asian populations (allele model: OR = 1.11, 95% CI: 1.04–1.19; homozygote model: OR = 1.22, 95% CI: 1.05–1.41; heterozygote model: OR = 1.21, 95% CI: 1.07–1.36; dominant model: OR = 1.21, 95% CI: 1.08–1.35), but not for Asian populations (Figure 3).

### 3.3. Publication Bias and Sensitivity Analysis

**Figure 4:**
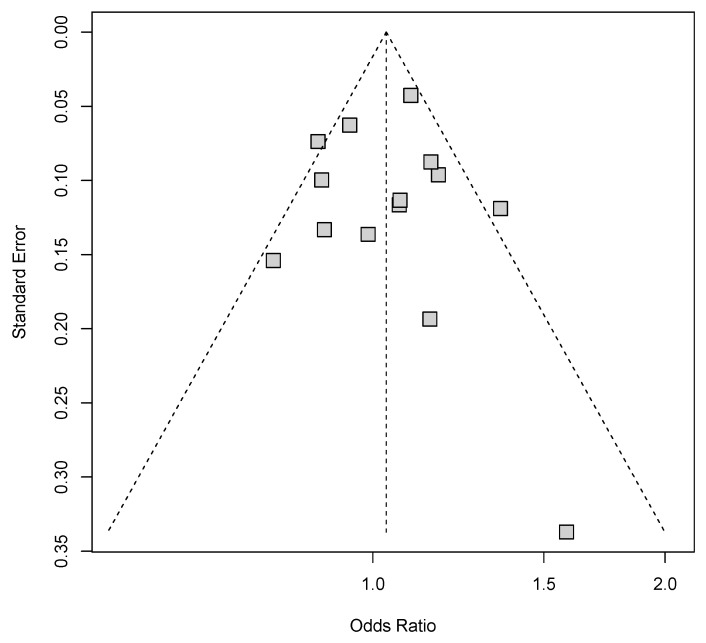
Funnel plot for publication bias under the allele model. Each gray square represents one single study involved in this meta-analysis.

Publication bias in all genetic models was graphically measured using the Begg’s funnel plots (Figure 4). No obvious evidence of asymmetric shape was observed. Further statistical assessment for the funnel asymmetry based on Egger’s test (allele model: *p* = 0.939; homozygote model: *p* = 0.745; heterozygote model: *p* = 0.943; dominant model: *p* = 0.947; recessive model: *p* = 0.403) and Begg’s test (allele model: *p* = 0.956; homozygote model: *p* = 1.000; heterozygote model: *p* = 0.784; dominant model: *p* = 0.411; recessive model: *p* = 0.626) revealed no significance. These results ruled out the possibility of publication bias.

**Figure 5:**
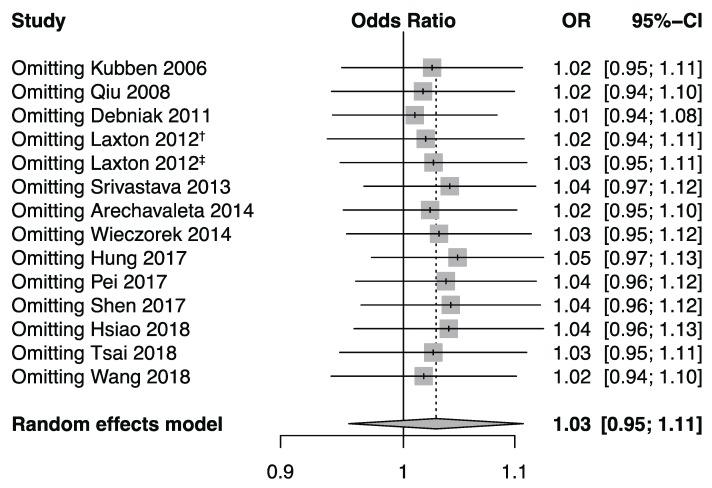
Forest plot for sensitivity analysis under the allele model. Pooled estimates were calculated using the random effects model. The named study was omitted to reappraise the association between the *MMP-8* rs11225395 polymorphism and cancer risk. † marks the population of the United Kingdom in Laxton’s study (2012), and ‡ marks the population of Poland in Laxton’s study (2012).

To verify whether the significance of the results was driven by any single data set, a sensitivity analysis was executed. The pooled estimates showed no statistical significance, which indicated no material change (Figure 5). These data evidenced the stableness of the results.

### 3.4. Between-Study Heterogeneity Analysis

**Figure 6:**
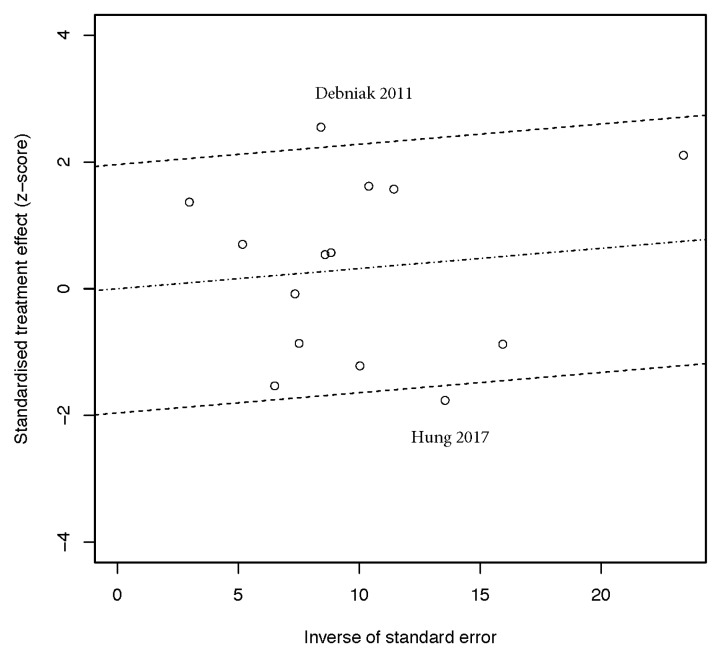
Galbraith plot for the source of between-study heterogeneity under the allele model. Each point represents one single study involved in this meta-analysis. The source of heterogeneity was marked by the author’s name and publication year.

To explore the source of between-study heterogeneity, we analyzed the statistics for heterogeneity in Galbraith plot analysis. Two data sets in the allele model [13,26], two in the homozygote model [13,31], two in the dominant model [13,26], and three in the recessive model [16,24,31] were identified as the sources leading to significant between-study heterogeneity (Figure 6). No significant heterogeneity could be detected after removing these data sets (allele model: *p* = 0.16; homozygote model: *p* = 0.25; dominant model: *p* = 0.22; recessive model: *p* = 0.63). Re-calculated pooled estimates under these genetic models remained stable (allele model: OR = 1.04, 95% CI: 0.99–1.09; homozygote model: OR = 1.03, 95% CI: 0.92–1.15; dominant model: OR = 1.06, 95% CI: 0.98–1.14; recessive model: OR = 1.00, 95% CI: 0.91–1.11).

## 4. Discussion

Meta-analysis is a requirement before the evidence for a pathogenesis association can be regarded as reliable. The current epidemiologic literature was systematically summarized to quantitatively assess the genetic impact of the *MMP-8* rs11225395 polymorphism on cancer risk. Based on the enlarged sample size and accumulated evidence, statistical power in the meta-analysis could be markedly enhanced, which consequently derives a more accurate and credible estimation. In this study, insignificant results were obtained for the impact of the *MMP-8* rs11225395 polymorphism on cancer susceptibility for the overall population. Similarly, no evidence of differential cancer risk in subgroups of cancer type was found. Neither the subgroups with large sample size nor those with small sample size revealed increased cancer risk. However, to our surprise, when stratified according to region, the non-Asian populations showed a significant association between elevated cancer risk and the T allele. The regional difference may be interpreted by the ethnic variance of genetic backgrounds, which could result in a synergistic interaction with other genetic factors. Haplotype risk estimation should be implemented when detailed genotype data for more loci are available. Environmental and lifestyle differences between regions should also be considered. Further adjustment for confounding factors, including cigarette smoking, alcohol consumption, environmental pollution, occupational exposure to hazardous chemicals, sanitary condition, and diet habits for the pooled estimates should be performed to evaluate their combined effect with this polymorphism when clinical information is ready.

This meta-analysis is useful to clearly realize the magnitude of the effect of rs11225395 on cancer risk. An important note of caution should be sounded, as the limited number of involved studies and relatively small sample size in both Asian and non-Asian subgroups may consequently lead to insufficient statistical power to determine the significance and potentially restrict the interpretation of the pooled estimates for cancer risk, in particular, concerning the influence of the risk variant on a specific type of cancer. The effect of rs11225395 on cancer susceptibility needs to be confirmed further. In addition, specific meta-analysis per tumor site should be performed individually to derive a more precise estimation of its genetic effects.

Significant between-study heterogeneity was identified in four genetic models. After removing the identified sources of heterogeneity from the Galbraith plot analyses, the statistic for heterogeneity showed no statistical significance, and the pooled estimates remained stable. These results illustrated the robustness of our conclusions.

A principal limitation is that our data sets search strategy was only used for databases in which the literature is in Chinese and English. Foreign languages, including French, German, and Japanese, were not taken into account due to our language limitation. Although the use of the five particular cyber databases in our systematic review provides significant data coverage security, additional data sets might have been retrieved if more databases had been queried. Furthermore, another primary limitation of this review is the statistically substantial heterogeneity for the results of pooled estimates, limiting the capability to precisely assess the size of the effects. Last but not least, analyzing rare events represents an inherent limitation because small variances in data could lead to material change for statistical significance. The use of relative measures of effect (e.g., OR) in meta-analysis could further exaggerate the instability of the results [36]. These limitations should be fully recognized before interpreting the results.

The main superiority of this systematic review is the methodology, including the literature search, study selection, information extraction, statistical analysis, and data interpretation, was rigorous. Most involved studies were identified to be of a good or moderate quality. Moreover, the advantage in sample size over a single case-control study led to the increased statistical power. Finally, the robustness and veracity of the pooled estimates were statistically ensured by our publication bias and sensitivity analyses. All these preponderances guaranteed the reliability of this meta-analysis.

## 5. Conclusions

Our results of this meta-analysis evaluating the relationship between the *MMP-8* rs11225395 polymorphism and cancer risk are reassuring, and suggest that this polymorphism is associated with elevated susceptibility to cancer in non-Asian populations. However, more large-scale case-control and prospective studies are needed to validate this population-specific correlation.

## Figures and Tables

**Table 1:** Principal characteristics of studies included in this meta-analysis.

Author	Year	Country	Region	Cancer Type	Matching Criteria	Genotyping Methods	Case-Control	HWE (*p*)	NOS
Kubben	2006	Dutch	Europe	gastric cancer	NA	PCR-RFLP	79-169	0.75	6
Qiu	2008	China	Asia	hepatocellular carcinoma	NA	PCR-RFLP	417-480	0.22	2
Debniak	2011	UK	Europe	melanoma	Age, gender	TaqMan	296-290	0.75	6
Hashim	2012	Malaysia	Asia	nasopharyngeal carcinoma	Age, gender	Microarray	48-48	NA	6
Laxton	2012	UK	Europe	breast cancer	Age	TaqMan	2172-2268	0.43	6
Laxton	2012	Poland	Europe	breast cancer	Age, gender	TaqMan	297-303	NA	5
Srivastava	2013	India	Asia	bladder cancer	Age, gender	PCR-RFLP	200-200	0.48	8
Arechavaleta	2014	Mexico	Latin America	ovarian cancer	NA	PCR-RFLP	35-37	0.01	5
Wieczorek	2014	Poland	Europe	bladder cancer	NA	TaqMan	241-199	0.70	5
Hung	2017	China	Asia	oral cancer	Age, gender	PCR-RFLP	788-956	<0.01	7
Pei	2017	China	Asia	leukemia	Age	PCR-RFLP	266-266	0.14	7
Shen	2017	China	Asia	lung cancer	Age, gender	PCR-RFLP	358-716	<0.01	8
Hsiao	2018	China	Asia	breast cancer	Age, gender	PCR-RFLP	1232-1232	<0.01	6
Tsai	2018	China	Asia	bladder cancer	Age, gender	PCR-RFLP	375-375	0.08	8
Wang	2018	China	Asia	breast cancer	NA	Mass spectrum	571-578	NA	7

UK: The United Kingdom. HWE: Hardy–Weinberg equilibrium. *p*-values for HWE of three data sets were marked with NA because the genotypes AA, Aa and aa were not obtained.

**Table 2:** Summarized genotype data included in this meta-analysis.

Author	Year	Country	Cancer								Control								
			CC	CT	TT	CC+CT	CT+TT	C	T	MAF	CC	CT	TT	CC+CT		CT+TT	C	T	MAF
Kubben	2006	Dutch	19	46	14	65	60	84	74	0.47	55	81	33	136	114		191	147	0.43
Qiu	2008	China	140	196	81	336	277	476	358	0.43	184	216	80	400	296		584	376	0.39
Debniak	2011	UK	86	152	58	238	210	324	268	0.45	113	134	43	247	177		360	220	0.38
Hashim	2012	Malaysia	NA	NA	1	47	NA	NA	NA	NA	NA	NA	8	40	NA		NA	NA	NA
Laxton	2012	UK	628	1105	439	1733	1544	2361	1983	0.46	735	1096	437	1831	1533		2566	1970	0.43
Laxton	2012	Poland	NA	NA	NA	NA	NA	332	262	0.44	NA	NA	NA	NA	NA		348	258	0.43
Srivastava	2013	India	99	90	11	189	101	288	112	0.28	92	84	24	176	108		268	132	0.33
Arechavaleta	2014	Mexico	6	16	13	22	29	28	42	0.60	6	26	5	32	31		38	36	0.49
Wieczorek	2014	Poland	72	125	44	197	169	269	213	0.44	60	101	38	161	139		221	177	0.44
Hung	2017	China	414	284	90	698	374	1112	464	0.29	466	364	126	830	490		1296	616	0.32
Pei	2017	China	139	98	29	237	127	376	156	0.29	129	105	32	234	137		363	169	0.31
**Shen**	2017	China	188	130	40	318	170	506	210	0.29	351	273	92	624	365		975	457	0.32
**Hsiao**	2018	China	648	466	118	1114	584	1762	702	0.28	633	468	131	1101	599		1734	730	0.30
**Tsai**	2018	China	186	152	37	338	189	524	226	0.30	197	140	38	337	178		534	216	0.29
**Wang**	2018	China	NA	NA	NA	NA	NA	724	418	0.37	NA	NA	NA	NA	NA		769	387	0.33

MAF: minor allele frequency (T/C+T).

**Table 3:** Association between the *MMP-8* rs11225395 polymorphism and cancer risk.

Comparison	Allele Model (T vs. C)			Homozygote Model (TT vs. CC)			Heterozygote Model (CT vs. CC)			Dominant Model (CT+TT vs. CC)			Recessive Model (TT vs. CC+CT)			
**(Number of Study)**	OR (95% CI)	*p*	*p_h_*	OR (95% CI)	*p*	*p_h_*	OR (95% CI)	*p*	*p_h_*	OR (95% CI)	*p*	*p_h_*	OR (95% CI)		*p*	*p_h_*
Overall (15)	1.03(0.95,1.11)	0.46	0.02	1.01(0.85,1.20)	0.90	0.03	1.06(0.98,1.14)	0.14	0.13	1.04(0.93,1.16)	0.49	0.03	0.97(0.83,1.13)		0.70	0.05
Region																
Asia (9)	0.97(0.89,1.07)	0.57	0.07	0.89(0.77,1.03)	0.12	0.18	0.97(0.88,1.07)	0.55	0.57	0.95(0.87,1.04)	0.26	0.34	0.89(0.77,1.02)		0.09	0.14
Others (6)	1.11(1.04,1.19)	<0.01	0.43	1.22(1.05,1.41)	0.01	0.39	1.21(1.07,1.36)	<0.01	0.43	1.21(1.08,1.35)	<0.01	0.45	1.09(0.96,1.24)		0.19	0.17
Cancer type																
Bladder (3)	0.97(0.83,1.12)	0.67	0.29	0.85(0.61,1.17)	0.32	0.14	1.08(0.87,1.33)	0.49	0.84	1.02(0.84,1.25)	0.81	0.58	0.83(0.61,1.12)		0.22	0.15
Breast (4)	1.06(1.00,1.13)	0.07	0.20	1.04(0.78,1.37)	0.80	0.08	1.08(0.89,1.30)	0.43	0.08	1.07(0.86,1.31)	0.55	0.04	1.02(0.89,1.16)		0.79	0.25
Digestive System (3)	1.03(0.83,1.28)	0.78	0.05	0.99(0.79,1.24)	0.95	0.11	1.10(0.80,1.49)	0.57	0.07	1.09(0.79,1.51)	0.59	0.04	0.97(0.79,1.20)		0.80	0.30
Others (5)	1.07(0.83,1.39)	0.60	0.02	1.14(0.71,1.86)	0.58	0.05	1.01(0.74,1.37)	0.96	0.09	1.03(0.75,1.44)	0.84	0.04	1.08(0.65,1.79)		0.78	0.02
Sample Size																
≤500 (5)	0.98(0.82,1.16)	0.80	0.20	0.87(0.60,1.27)	0.48	0.11	1.08(0.83,1.41)	0.56	0.46	1.02(0.79,1.31)	0.88	0.51	0.82(0.41,1.64)		0.57	<0.01
>500 (10)	1.03(0.95,1.12)	0.41	0.01	1.03(0.87,1.23)	0.71	0.04	1.05(0.93,1.18)	0.43	0.06	1.04(0.92,1.19)	0.52	<0.01	1.01(0.91,1.11)		0.86	0.44

OR: odds ratio. CI: confidence interval. *p_h_*: *p*-value for between-study heterogeneity.
